# Physicochemical, steric, and energetic characterization of kaolinite based silicate nano-sheets as potential adsorbents for safranin basic dye: effect of exfoliation reagent and techniques

**DOI:** 10.3389/fchem.2024.1455838

**Published:** 2024-10-18

**Authors:** Samar Mohamed Ali, Reham A. Mohamed, Ahmed A. Abdel-Khalek, Ashour M. Ahmed, Mostafa Abukhadra

**Affiliations:** ^1^ Department of Chemistry, Faculty of Science, Beni-Suef University, Beni-Suef, Egypt; ^2^ Materials Technologies and their Applications Lab, Geology Department, Faculty of Science, Beni-Suef University, Beni-Suef, Egypt; ^3^ Physics Department, College of Science, Imam Mohammad Ibn Saud Islamic University (IMSIU), Riyadh, Saudi Arabia; ^4^ Geology Department, Faculty of Science, Beni-Suef University, Beni-Suef, Egypt

**Keywords:** kaolinite, exfoliation, intercalating agent, safranin, adsorption, advanced equilibrium

## Abstract

Kaolinite was subjected to advanced exfoliation processes to form separated nano-silicate sheets (EXK) with enhanced physicochemical properties as adsorbents. This involved the incorporation of different exfoliating agents, urea (U/EXK), KNO_3_ (N/EXK), and CTAB (C/EXK), highlighting their impacts on their textural and surficial properties as adsorbents for safranin dye. The applied characterization techniques confirmed the higher exfoliating degree of C/EXK, followed by N/EXK and U/EXK. This appeared significantly in the determined surface area (55.7 m^2^/g (C/EXK), 36.7 m^2^/g (U/EXK), and 47.1 m^2^/g (N/EXK)) and adsorption performances. The C/EXK structure displayed a better safranin uptake capacity (273.2 mg/g) than N/EXK (231 mg/g) and U/EXK (178.4 mg/g). Beside the remarkable differences in textural properties, the advanced mathematical modeling and the corresponding steric and energetic parameters illustrate the mentioned uptake properties. The interface of C/EXK is highly saturated by active uptake sites (Nm = 158.8 mg/g) as compared to N/EXK (109.3 mg/g) and U/EXK (93.4 mg/g), which is in agreement with the characterization findings and the expected higher exposure of siloxane groups. Each of these sites can be filled with four dye molecules using C/EXK and N/EXK, which implies the vertical orientation of these adsorbed ions and the effective operation of multi-molecular mechanisms. The energetic (ΔE < 40 kJ/mol) and thermodynamic investigations indicate the spontaneous, physical, and exothermic uptake of safranin molecules by EXK particulates. These mechanisms might involve dipole bonding (2–29 kJ/mol), electrostatic attraction (2–50 kJ/mol), van der Waals forces (4–10 kJ/mol), and hydrogen bonding (<30 kJ/mol).

## 1 Introduction

The primary peril facing the contemporary world is the pollution of potable water as well as the safeguarding of its populace (Yang et al., 2022; Zourou et al., 2022). The World Health Organization (WHO) issued a dire warning, predicting that by 2025, almost half of the world’s population would experience substantial water scarcity (Zourou et al., 2022; Arab et al., 2022). The exponential growth of the industrial sector within the past century has resulted in significant environmental issues, such as water contamination, which has had adverse impacts on human beings alongside aquatic ecosystems (Yang et al., 2022; Jawad et al., 2022). Industrial activities release several water pollutants, including bacteria, pesticides, hazardous metals, pharmaceutical residuals, fertilizers, and dyes (Hassan et al., 2022; Prajapati and Mondal, 2022). Synthetic dyes are a varied range of aromatic compounds that are extensively used as crucial coloring agents for numerous industries, including plastic, leather, paper, and textiles (Amri et al., 2023; Bensalah et al., 2023). As a result, an estimated amount exceeding 700,000 tons of the synthesized dyes gets released annually into the surrounding areas and aquatic ecosystems (Kadiri et al., 2021).

The majority of chemically synthesized dyes are toxic and resistant to biological degradation, leading to harmful impacts on both ecosystems and human health (Arab et al., 2022; Kadiri et al., 2021). Safranin dye (SF) is a type of water-soluble basic azine dye that is commonly used in textiles for coloring applications (Laskar and Kumar, 2023; El-Sherbeeny et al., 2021). It is also utilized in various other applications, such as stains, recognizing microorganisms, the healthcare sector, and the packaging of foods (de Araujo et al., 2023; Sarkar et al., 2024). The complex structure and stability of SFR provide difficulties during its biological degradation (de Araujo et al., 2023). This dye has the ability to destroy the nucleic acid of bacteria as well as exhibit tumor-promoting and mutagenic properties. Prolonged or short-term contact with SFR may result in a variety of negative health effects, including irritation of the lips, eyes, stomach, and tongue, as well as itching and redness of skin surfaces. Additional symptoms associated with SFR exposure involve nausea, emesis, and gastrointestinal distress (Shaltout et al., 2022; El-Sherbeeny et al., 2021).

Therefore, the concentrations of SFR, as most synthetic dyes recommend, should be lower than 1 mg/L in the drinking water. Consequently, many techniques have been developed for eliminating dye contaminants, considering the aforementioned concerns about human wellbeing and the environment. The techniques involve ozonation (Le et al., 2022), photocatalytic oxidation (Roy and Chakraborty, 2021), flocculation/coagulation (Basaleh et al., 2021), co-precipitation (Ashrafi et al., 2022), adsorption (Jebli et al., 2023), and ion exchange (Lahiri et al., 2022). The adsorbing method is widely supported as an effective and affordable method to eliminate dyes. This technique has numerous advantages, including high elimination efficiency, a wide variety of applications, simple reuse and recycling abilities, and cost-effective production methods (Lebkiri et al., 2023; Pandey et al., 2022). Hence, researchers have investigated several mono and hybrid structures to provide efficient potential adsorbents for dyes (Zourou et al., 2022; Arab et al., 2022; Kenawy et al., 2022). However, the selection of suitable adsorbents is controlled by several factors, including availability, synthesis cost, adsorption efficiency, adsorption selectivity, recovery, and recyclability (Abukhadra et al., 2022a).

Therefore, the newest research investigation tested and developed several synthetic adsorbents derived from the earth’s resources. These types of adsorbents have proven to be remarkably effective and favorable in removing various types of organic contaminants, including dyes, from water, whether in their pure form or in blend with other materials (Ahrouch et al., 2019). Recent investigations focused on innovative modified types of synthesized clay minerals as cost-effective, efficient, and environmentally friendly solutions for decontaminating both organic and inorganic soluble chemicals (Ahrouch et al., 2022; Salam et al., 2020). The majority of recognized clay minerals exhibit adaptable and reacting layered aluminosilicate chemical frameworks that possess notable ion exchange characteristics, chemical reactivity, biological compatibility, active surface, excellent adsorption capacities, natural abundance, nontoxic nature, and thermal resistance (Ahrouch et al., 2020; Dardir et al., 2018). Kaolinite is a naturally occurring mineral with a framework composed of hydrous aluminum silicate layers. These layers are composed of layered tetrahedron/octahedron subunits at a 1:1 ratio (Tian et al., 2020; Shaban et al., 2018). Nevertheless, despite its abundant deposits, accessibility, and cost-effectiveness in comparison to other frequently utilized clay minerals, kaolinite has limited uses in both environmental and commercial industries (Tian et al., 2020; Carretero and Pozo, 2010). Some of the reported limitations for the application of kaolinite include its relatively small surface area and poor ion exchange ability in comparison to frequently employed clay minerals such as halloysite and montmorillonite (Abukhadra and Allah, 2019).

Consequently, various approaches were employed to improve the physicochemical characteristics of kaolinite, including organic and inorganic modifications, exfoliation, and scrolling (Tian et al., 2020; Shaban et al., 2018; Abukhadra and Allah, 2019). Throughout the past few years, there has been notable advancement in the modification and kaolinite conversion methods, which involve exfoliating the layered clay into separated silicate sheets that have two-dimensional morphologies (Zhang et al., 2017; Allah et al., 2023). The implementation of this technique resulted in the development of new nanostructures comprised of clay units, which possess significant characteristics such as impressive biological safety, adsorption capability, surface area, oxidation characteristics, reacting surface features, anti-cancer activity, and dispersion characteristics (Tian et al., 2020; Abukhadra M. R. et al., 2020; Zuo et al., 2017). Common exfoliation techniques comprise high-pressure extrusion, sonication, chemical intercalation, physical grinding, and others (Zuo et al., 2017; Alqahtani et al., 2023). Chemical intercalation methods have been recognized as the best and most efficient method to accomplish the peeling and exfoliation of kaolinite (Zhang et al., 2017; Zuo et al., 2017). The intercalated exfoliating chemicals included N-methylformamide, dimethylsulfoxide, urea, alkylamines, potassium acetate, formamide, fatty acids, quaternary ammonium salts, and hydrazine hydrate (Zuo et al., 2017; Makó et al., 2019). Prior research has shown that various organic guest types, whenever inserted between the kaolinite layers, not only significantly expand the dimension of the interlayer gap but additionally substantially destruct the hydrogen bonding within the kaolinite-layered units. Enhanced exfoliating of kaolinite-layered units is facilitated by reduced interfacial adhesion (Makó et al., 2019; Abdo et al., 2022).

The structural, morphological, textural and physicochemical characteristics of the exfoliated kaolinite as well as the exfoliation efficiency are strongly influenced by the intercalating chemical agents used (Makó et al., 2019; Abdo et al., 2022; Shawky et al., 2019) Unfortunately, few studies and, to our knowledge, no previous studies have highlighted the impact of the applied exfoliating agents and techniques on the efficiency of the obtained separated silicate sheets as well as their physicochemical and adsorption properties. It was expected that the exfoliating techniques would significantly affect the morphological, textural, physicochemical, and adsorption properties of the end product. This strongly endorses the conduct of extensive comparative studies that highlight the impact of the exfoliation methodologies and the used agents on the separated silicate sheets, particularly as adsorbents, considering the interaction at the adsorbate/adsorbent interface. Therefore, the presented study aims to evaluate the impact of three intercalating agents (CTAB, potassium nitrate, and urea) or their exfoliation techniques on the physicochemical and adsorption properties of exfoliated kaolinite (C/EXK, N/EXK, and U/EXK) as potential adsorbents for safranin-O synthetic dye. These involved experimental investigation of the affecting factors in addition to extensive equilibrium modeling, either by classic models or advanced models. The advanced models were designed based on statistical physics theory, and the main parameters involved existing effective site density, capacity of each uptake site, saturation capacity, and energetic and thermodynamic factors.

## 2 Experimental work

### 2.1 Materials

The kaolinite powder (KA) used to produce the exfoliating layers (EXK) was obtained from the Central Metallurgical and Development Institute in Egypt. Chemically, the KA sample composed of SiO_2_ (47.83%), Al_2_O_3_ (35.74%), Fe_2_O_3_ (0.89%), MgO (0.12%), CaO (0.53%), TiO_2_ (0.82%), Na_2_O (0.28%), K_2_O (0.08%), and loss of ignition (14%). The initial exfoliating approach involved using dimethyl sulfoxide (DMSO) (CAS: 67-68-5) that had a quality exceeding 99.5%, CTAB (cetyltrimethylammonium bromide) (CAS: 57-09-0) with a quality over 98%, and methanol of a quality exceeding 99.9% (CAS: 67-56-1; Sigma-Aldrich). Urea (U) with a purity of 99% from Sigma-Aldrich and potassium nitrate (KNO_3_) with a purity of 98% have been employed during the exfoliating processes. NaOH, HNO_3_, and N_4_OH solutions having particular concentrations were implemented in a variety of processing and adjustment approaches without undergoing purification. The adsorption experiment was conducted employing safranin-O synthetic dye (≥85%; CAS: 477-73-6; Sigma-Aldrich) as the main source of synthesized dye pollutants.

### 2.2 Exfoliation of kaolinite

#### 2.2.1 Exfoliation by CTAB induced technique (C/EXK)

**FIGURE 1:**
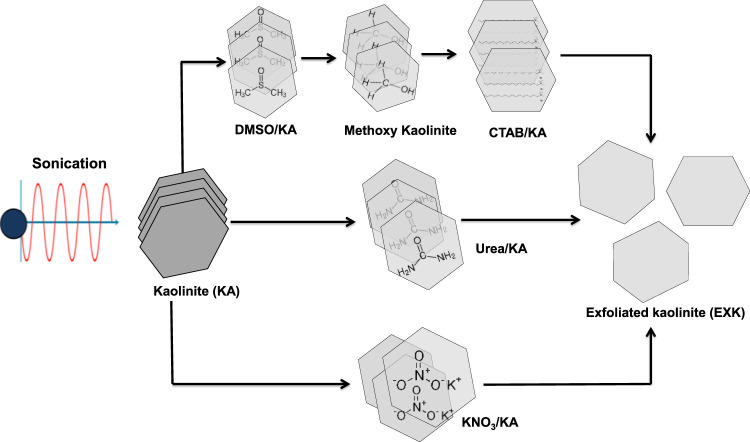
Schematic diagram for the synthesis of exfoliated kaolinite using different intercalating agents.

The kaolinite-layered subunits were exfoliated by a simple chemical-based expansion approach. The original kaolinite sample underwent a pulverizing step for 6 h inside a ball mill, leading to the development of kaolinite granules with particle diameters ranging between 20 and 100 μm. The pulverized kaolinite (15 g) was then thoroughly mixed with 50 mL of DMSO (8 DMSO: 1 water) over a period of 5 h using a conventional magnetic stirrer device. The step described earlier is essential for the destruction of the existing hydrogen bonding connecting the layered silicate units. The kaolinite, which had been immersed in DMSO, was thereafter exposed to a series of washings using methanol for an interval of 20 min each. The process as described was iterated five runs to remove the encapsulated DMSO molecules and replace them with methanol. Consequently, a methoxy-modified kaolinite (Mth/K) had been developed. The Mth/K suspended particulates underwent a homogenizing step with CTAB solution (20 g CTAB + 50 mL distilled water). This step continued over 48 h and encompassed intricate mixing apparatus, which comprises a magnetic stirrer along with an ultrasound generator (240 W). This process resulted in the development of exfoliating or separated kaolinite flakes (EXK). Subsequently, the resultant EXK particulates underwent extensive washing implementing distilled water and then underwent a slow drying step at a temperature of 65°C for an interval of 12 h (Figure 1). The dried particulates were identified as C/EXK and preserved for further characterization and application procedures.

#### 2.2.2 Exfoliation by urea (U/EXK) and KNO_3_ (N/EXK)

The exfoliating processes employing the two chemicals were performed according to the methodology described by Shawky et al. (2019). A total of 3 g of finely powdered kaolinite was combined with 1.5 g of urea by utilizing an agate mortar over a duration of 15 min. The mixture was then subjected to thermal treatment for 48 h employing an electric computerized muffle furnace at a temperature of 95°C. For the KNO_3_-prepared product, 3 g of the powdered KA precursor had been homogeneously dispersed throughout a water-based solution of KNO_3_ (60 mL) over 48 h at 90°C employing a magnetic stirring device (500 rpm). The KA particulates, which had been embedded with the urea alongside KNO_3_, were then separated by filtering, underwent a thorough washing process utilizing distilled water through three cycles (each extending 10 min), and subsequently rinsed with ethanol. The washed materials were then subjected to a mild drying procedure for 24 h at a temperature of 60°C. In order to accomplish the successful splitting of the KA particulates into independent layers, the embedded materials thereafter underwent an extra heat-treatment process for a duration of 60 min at a temperature of 120°C. Lastly, the exfoliating products were designated as U/EXK and N/EXK after applying urea and KNO_3_, respectively (Figure 11), and preserved for the subsequent characterization and examination stages.

### 2.3 Analytical techniques

The PANalytical-Empyrean X-ray diffractometer was used to ascertain the degree of crystallization and the corresponding crystalline varieties. The diffraction patterns were determined across the range of 0°–70°. The chemical structures of C/EXK, U/EXK, and N/EXK were determined with a Fourier transform infrared spectrometer (FTIR8400S; Shimadzu) through a range of frequencies from 400 cm^−1^ to 4,000 cm^−1^. The Gemini Zeiss Ultra 55 scanning electron microscope was used to obtain the SEM photos of the evaluated structures after depositing slim gold covers over their surfaces. These photos were used to track the expected changes in morphology resulting from the three exfoliating techniques. In addition, the interior features of C/EXK, U/EXK, and N/EXK have been further investigated using high-resolution HRTEM photos obtained from a JEOL-JEM2100 transmission electron microscope operating at an accelerated voltage equal to approximately 200 kV. The surface area and porosity of C/EXK, U/EXK, and N/EXK were evaluated using a Beckman Coulter SA3100 analyzer in conjunction with the relevant N_2_ adsorption/desorption isotherms.

### 2.4 Adsorption studies

#### 2.4.1 Batch adsorption studies

The adsorption capacities of C/EXK, U/EXK, and N/EXK as adsorption agents towards safranin-O dye (SFR) were successfully investigated by a series of batch uptake tests. The investigation comprised several influential factors, such as pH values spanning from 3 to 8, starting dye concentrations that varied from 25 to 300 mg/L, and uptake periods varying from 15 to 1,440 min. The overall volumes of contaminated solutions and the amounts of C/EXK, U/EXK, and N/EXK were kept consistent at 100 mL and 0.2 g/L, respectively. Nevertheless, the temperature at which the adsorption occurred varied from 303 K to 323 K for the whole investigation. The mixing process during the adsorption was performed using orbital shaker incubator swathe digital temperature controller. Following the end of all assessments, the residual dye was measured using a UV-Vis spectrophotometer at a detection wavelength of 521 nm. The dye levels were monitored to determine the adsorption capacities of C/EXK, U/EXK, and N/EXK. Equation 1 was used to calculate the result, implementing the treated volume (V), dosage (m), initial level (C_o_), and leftover level (C_e_).

$$Q_{e\left(\text{mg}/g\right)}=\frac{\left(C_{O}-C_{e}\right)V}{m}$$ (1)

#### 2.4.2 Theoretical traditional and advanced equilibrium studies

The simulation of adsorption behaviors has been accomplished using classic kinetic, normal isotherm, and advanced forms of equilibrium models based on statistical physics hypotheses (Supplementary Table S1). Nonlinear fitting methods were used to fulfill the kinetic and classic equilibrium simulations, utilizing the mathematical formulations for these models. The obtained results of the correlation coefficient (*R* ^2^) (Equation 2) together with Chi-squared (χ^2^) (Equation 3) were subsequently used to detect the fitting degrees. The compatibility of the adsorption activities with the evaluated advanced equilibrium theories has been verified by implementing the coefficient of correlation (*R* ^2^) and the root mean square error (RMSE) (Equation 4). The symbols m′, p, Qi_cal_, and Qi_exp_ represent the actual observations, investigated variables, expected SFR adsorption, and proved adsorption effectiveness, respectively.

$$R^{2}=1-\frac{\sum {\left(Q_{e,\exp}-Q_{e,\text{cal}}\right)}^{2}}{\sum {\left(Q_{e,\exp}-Q_{e,\text{mean}}\right)}^{2}}$$ (2)

$$\chi^{2}=\sum \frac{{\left(Q_{e,\exp}-Q_{e,\text{cal}}\right)}^{2}}{Q_{e,\text{cal}}}$$ (3)

$$\text{RMSE}=\sqrt{\frac{\sum_{i=1}^{m}{\left({\text{Qi}}_{\text{cal}}-{\text{Qi}}_{\exp}\right)}^{2}}{m^{\prime }-p}}$$ (4)

## 3 Results and discussion

### 3.1 Characterization of the adsorbent

#### 3.1.1 XRD analysis

**FIGURE 2:**
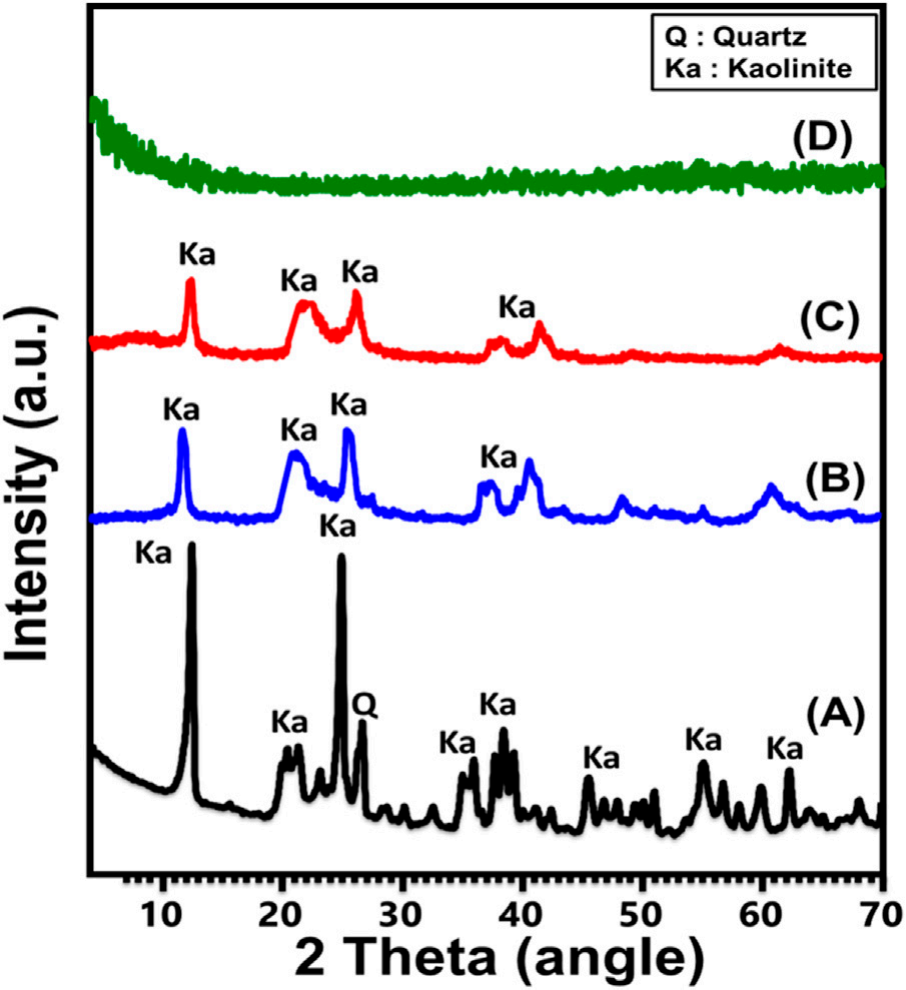
XRD patterns of raw kaolinite **(A)**, U/EXK **(B)**, N/EXK **(C)**, and C/EXK **(D)**.

The crystalline modifications that occur throughout the transformation of KA mineral into individual and exfoliated nano-KA layers (EXK) by various intercalating agents have been monitored utilizing their XRD patterns (Figure 1). The well-defined peaks around angles of 12.33° (001) and 24.87° (002) (Figure 2A) identify the triclinic, highly crystalline KA mineral as the initially implemented crystallized component. The kaolinite’s crystallization index (HI) is described by its less intense peaks occurring inside the Hinckley range (H), which extends from 12.33° to 24.87°. The basal-spacing distance correlating with these diffraction peaks was detected at 0.72 nm. Regarding the prepared EXK utilizing urea (U/EXK) (Figure 2B) and KNO_3_ (N/EXK) (Figure 2C) as intercalating agents, the obtained patterns demonstrate significant structural changes but without extensive destruction of the crystalline structure. The patterns of U/EXK and N/EXK display a notable reduction of the essential peaks of KA and extensive declination in the strengths of Hinckley index peaks, indicating the successful exfoliation of the layered units without extensive destruction of the crystalline structure or its partial exfoliating impacts. Moreover, considering the reduction degress in the main peaks as well as the Hinckley index peaks, it suggests the higher efficiency of the KNO_3_-based exfoliating process than the urea-based processes. Finally, the patterns reflected no indication about the intercalated compound, i.e., the structure is free of the used exfoliating agents.

The diffraction pattern obtained after the sonication-supported exfoliation process employing CTAB (C/EXK) displayed a total elimination of the strength of all remaining peaks, suggesting that the resulting material had an amorphous or semi-crystalline structure (Figure 2D). This confirms the complete destruction of the crystal system and the extensive exfolation of the KA-layered units into separated and distinct silicate layers. The previous findings declared the higher performances of the CTAB intercalating method on the exfolaiting efficacy of the kaolinite as compared to the application of the other two methods.

#### 3.1.2 SEM and HRTEM analyses

**FIGURE 3:**
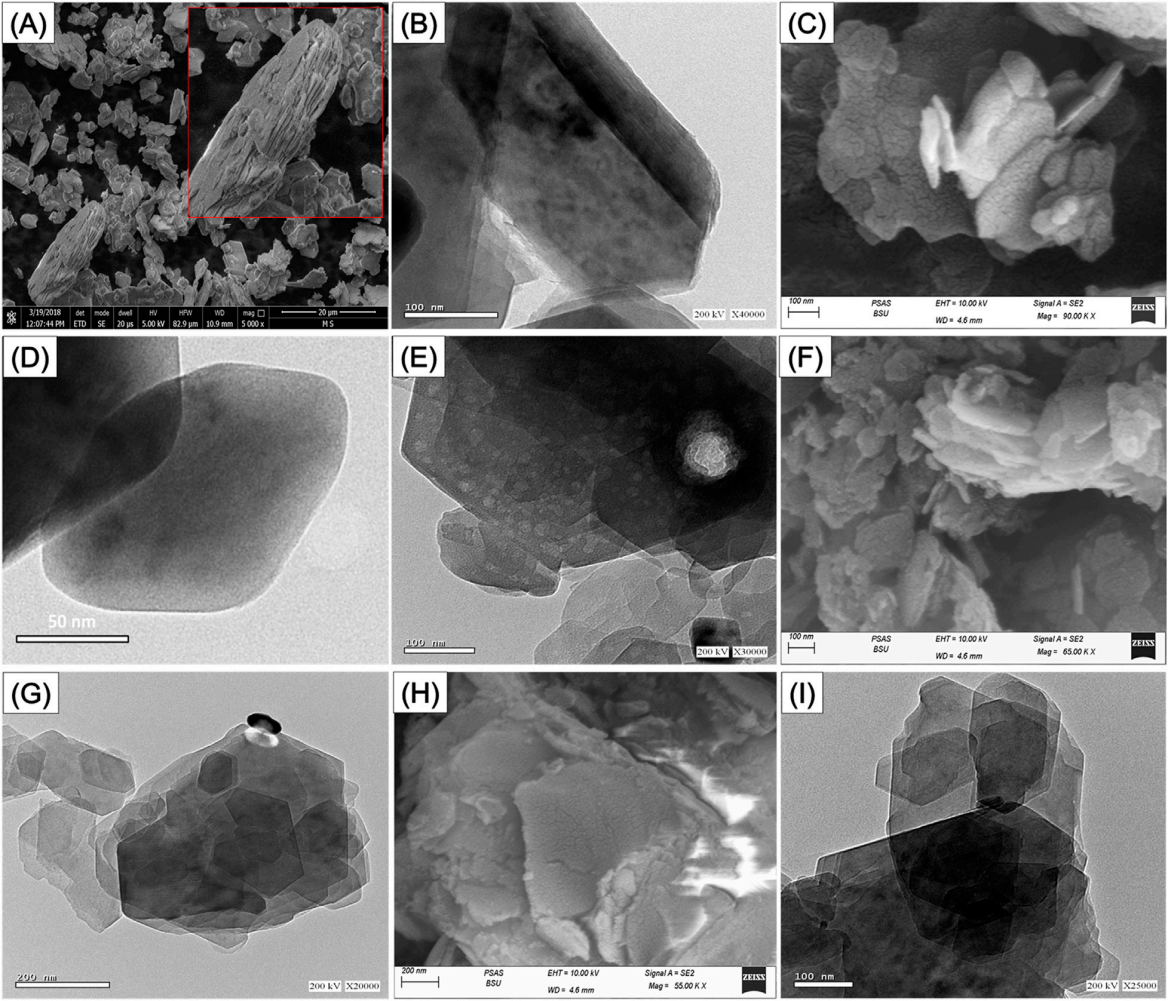
SEM of raw kaolinte **(A)**, HRTEM of raw kaolinite **(B)**, SEM of synthetic C/EXK **(C)**, HRTEM images of C/EXK **(D, E)**, SEM image of N/EXK **(F)**, HRTEM image of N/EXK **(G)**, SEM of U/EXK **(H)**, and HRTEM image of U/EXK **(I)**.

To follow the morphological and geometrical impacts of the applied exfollation techniques in correlatin with used intercalating agents, the SEM and HRTEM images of the raw KA as well as C/EXK, U/EXK, and N/EXK were inspceted (Figure 3). The starting precursor displays the known geometries and morphology of the highly crystalined KA mineral as flakes or plate-like grains of pesudo-hexagonal outlines that are stacked above each other in aggeragtes or agglomerated form, either in the SEM (Figure 3A) or HRTEM (Figure 3B) photos. Regarding the exfolaikted KA based on CTAB intercalation (C/EXK), the recognized photos demonstrate very effective stripping and splitting of the structural KA layered units into discrete and separated sheets (Figures 3C–E). The sperated sheets still exhibit relict pseudo-hexagonl geometry, but with notable smooth outlines or borders in contrast to the raw particulates (Figure 3D). Some inspected exfolaited grains show batches of a milder gray shade in contrast to the general gray shade of KA flake, suggesting distortion in the ordering properties of the structural silicate units of KA (Figure 3E).

The analyzed photos of both U/EXK and N/EXK also demonstrate the successful separation of the kaolinite units into discrete sheets, but with a lower degree as compared to C/EXK, which is in agreement with the XRD indications. The exfoliation was observed by the reduction in the stacking degree of the KA layers and the declination in the dimension of the present falke-like grains. Moreover, the application of KNO_3_ as an intercalating agent during the exfoliating process (N/EXK) (Figures 3F,G) appeared to be more effective than urea (U/EXK) (Figures 3H,I). The recognized particulates of N/EXK shower lower stacking degrees and more discerer sheets than U/EXK particulates. Furthermore, milder gray batches can be detected in the KNO_3_-based exfolaited particles, in addition to the noticeable changes in the pseudo-hexagonal outlines. Such obsrevation reflected higher dsirtorytion in the structure of the KA after its modification with KNO_3_ during the exfoliation as compared to urea, as concluded by the XRD investigation.

#### 3.1.3 FT-IR analysis

**FIGURE 4:**
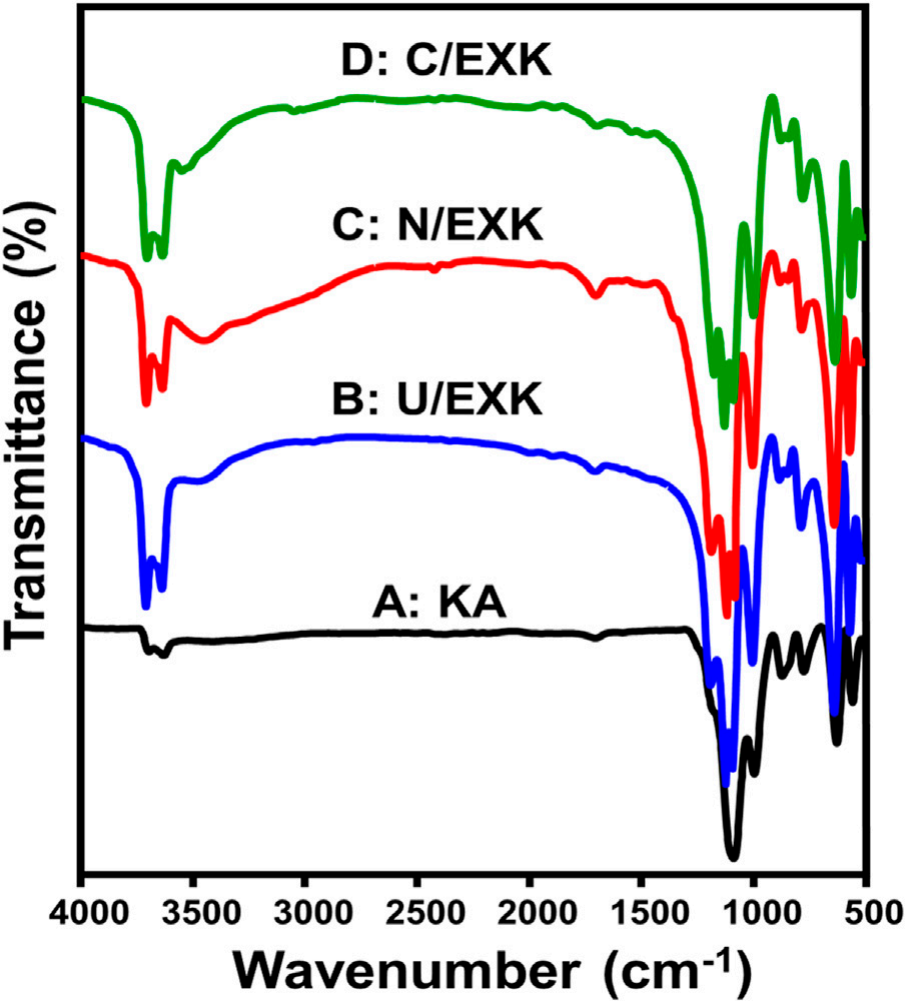
FT-IR spectra of raw kaolinite **(A)**, U/EXK **(B)**, N/EXK **(C)**, and C/EXK **(D)**.

The FT-IR spectra have been employed to assess the impact of the employed intercalating agents or exfoliation techniques on existing functional chemical groups. The spectrum of KA displays clearly identifiable bands that correlate to the distinctive functional units detected throughout its aluminosilicate framework as a clay mineral (Figure 4). These involve Si-O (787 and 456 cm^−1^), Si-O-Al (526 and 680 cm^−1^), Si-O-Si (1,020 cm^−1^), Al-OH (912 and 3,500 cm^−1^), O-H (1,641 cm^−1^), and Si-OH (3,689 cm^−1^) (Figure 4A) (Tian et al., 2020; Adly et al., 2022). The spectra of U/EXK (Figure 4B), N/EXK (Figure 4C), and N/EXK (Figure 4D) display absorbing bands which are similar to those observed for untreated KA. However, there are notable differences in their positions, reductions in their intensity, and the splitting of the distinct bands centered at 900 cm^−1^ and 1,000 cm^−1^. This signifies the effective separation of the KA forming aluminosilicate sheets, leading to the development of single-layered structures or separate nanosheets. Furthermore, the exfoliating processes induce considerably the distortions of both the octahedron and tetrahedron layered units (Abukhadra and Allah, 2019; Shawky et al., 2019). The investigated samples exhibit shifts in the positions of the bands caused by the breakdown of hydrogen bonding within successive KA layers. Additionally, there were variations in strength and widening of the inspected bands, which can be attributed to the development of newly formed hydrogen bonds among KA interlayers together with the functionalities of embedded inercalating chemicals. These findings provide evidence that the interior OH groups of KA are reactive and not inert (Zsirka et al., 2016). Additionally, the remerakable intensification of the coresponding bands of the active siloxane groups demonstrates the effective impact of the exfaolaition processes in inducing and enhancing the exposure of such active groups.

#### 3.1.4 Textural analysis

**FIGURE 5:**
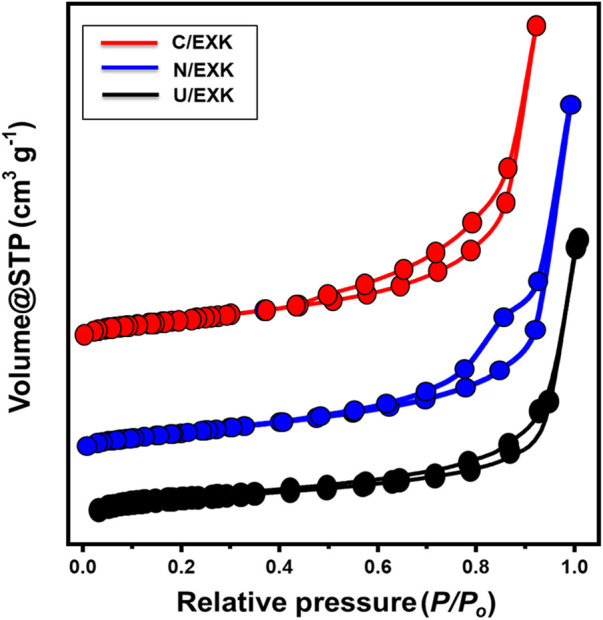
The N_2_ adsorption/desorption isotherm curves of U/EXK, N/EXK, and C/EXK.

The N_2_ adsorption/desorption isotherms were applied to assess the KA, C/EXK, U/EXK, and N/EXK exterior textural characteristics. The isotherm curves for C/EXK, U/EXK, and N/EXK have been identified as IV-type curves that include detectable H3 hysteresis loops. This kind was often described in relation to the potential capillary condensation-induced evacuating or filling of mesopores exhibiting tabular or cylindrical geometries (Figure 5). The observed surface area clearly improved using the various exefoliation techniques, as seen by the derived curves. The measured surface areas are 10 m^2^/g, 36.7 m^2^/g, 47.1 m^2^/g, and 55.7 m^2^/g for the KA, U/EXK, N/EXK, and C/EXK, respectively. Such values are in agreement with the previously obtained results from the XRD and SEM investigations. The exfoliating modifications based on the CTAB-induced sonication techniques resulted in effective separations of the kaolinite units into monosheets or nano-silicate layers as compared to the applied techniques based on the application of urea and KNO_3_ as intercalating agents. Regarding the impact of the applied techniques on the porous properties of the obtained exfoliated products, the determined average pore volumes for KA, U/EXK, N/EXK, and C/EXK are 0.052 cm^3^/g, 0.106 cm^3^/g, 0.248 cm^3^/g, and 0.32 cm^3^/g, respectively. This increase in the pore volumes suggested a significant impact for the intercalating agent, inducing basal spascing, the exspour of structural hexagol molecular pores within the silicate tetrahedron sheets, and the expected bending of the silicate sheets during the expansion effect of the intercalating agents, which can be detected clearly in the C/EXK particles. Such changes are also associated with considerable changes in the pore size and dirtibution properties. The pore diameter for all the samples spanned from 2 nm up to about 11 nm, with the average diemater equal to 6.4 nm for U/EXK, 5.5 nm for N/EXK, and 5.12 nm for C/EXK. Such values declare the exfoliation of the keolinite as a mesoporous material, which is favorable during adsorption applications.

### 3.2 Adsorption results

#### 3.2.1 Effect of the pH

**FIGURE 6:**
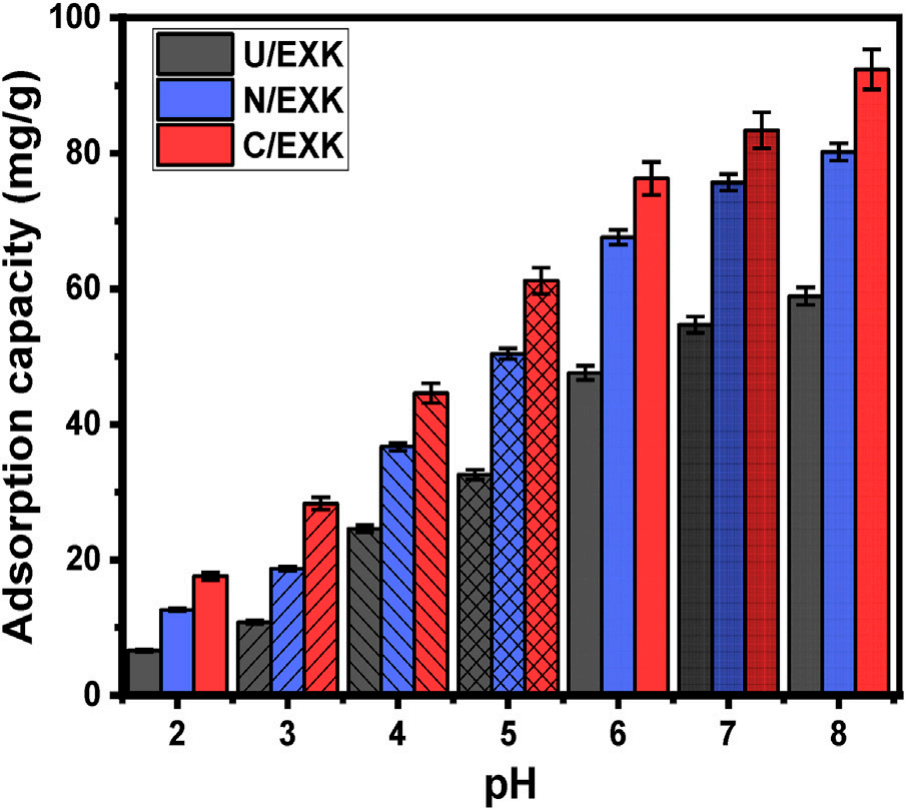
The influence of pH on the adsorption of SFR by U/EXK, N/EXK, and C/EXK.

As it affects the adsorbent’s external charges alongside the ionization behavior of dissolved pollutants in water-based solutions, the pH factor has significance during the adsorption reaction. The adsorption characteristics of U/EXK, N/EXK, and C/EXK towards the removal of SFR were investigated in terms of the changes in pH. The study comprised a pH range of 2–8 and maintained other specified values at 100 mL for the beginning volume, 100 mg/L for the SFR concentration, 120 min for the study’s duration, 0.4 g/L for the dosage, and 20°C for the assessment temperature. The adsorption results were computed as averaged values using the results from three separate tests, and the standard deviations are below 4.2% for all the tests. As can be determined by the findings of determining the amounts of SFR adsorbed using U/EXK, N/EXK, and C/EXK, the results showed a noticeable rise in SFR uptake as frequently the pH value of the analyzed polluted solutions progressed over pH 3 (Figure 6). From 6.6 mg/g (U/EXK), 12.6 mg/g (N/EXK), and 17.2 mg/g (C/EXK) at pH 2–58.9 mg/g (U/EXK), 80.2 mg/g (N/EXK), and 92.4 mg/g (C/EXK) at pH 8, retention qualities improved (Figure 6). In accordance with the pH specifications for industrial wastewater remediation established by the US EPA (pH 6–9), together with the measured efficacy of the evaluated structures throughout various pH levels, the examined structures could therefore be deemed adequate to be employed as reliable adsorbents during realistic treatment for SFR (Vivas and Cho, 2021). This included the elimination of SFR from the industrial wastewater corresponding to staining industries as well as the healthcare sector and the packaging of foods. The uptake activities can be illustrated based on the ionizing activity of SFR as a basic dye in conjunction with the dominant surface charges throughout the U/EXK, N/EXK, and C/EXK structures.

In terms of SFR’s ionizing characteristics, the SFR molecules possess positive electrical charges as a result of its protonation behavior at pH < 11 for its pKa value (pKa = 11). Therefore, the soluble SFR molecules at the highest level of studied pH values (pH 8) display powerful electrostatic attractive forces with the deprotonated and negative-charged functionalities of U/EXK, N/EXK, and C/EXK at this pH level (basic environment) (Adly et al., 2022; Vivas and Cho, 2021; Sriram et al., 2024). Moreover, at a significantly low pH, the structural amino group (-NH_2_) of safranin undergoes protonation as a result of the high level of hydrogen ions that exist inside the dye solution. This protonation hinders the development of hydrogen-bonding between the SFR molecules and the interface of adsorbents (Ghosh et al., 2021). Also, this induces the formation of electrostatic repulsive forces between the dye molecules with the protonated and positively charged surfaces of EXK particles (Ghosh et al., 2021). By analyzing the optimal pH value in comparison with the values of pH_PZC_ that were measured for the adsorbents (6.8 (U/EXK), 6.6 (N/EXK), and 7.3 (C/EXK), the adsorptive elimination of SFR provided higher efficiency beyond the pH_PZC_. In general, the EXK structures possess negative surface charges when the pH is higher than pH_PZC_, but they turn positive when the pH is lower than pH_PZC_.

#### 3.2.2 Kinetic studies

##### 3.2.2.1 Effect of contact time

**FIGURE 7:**
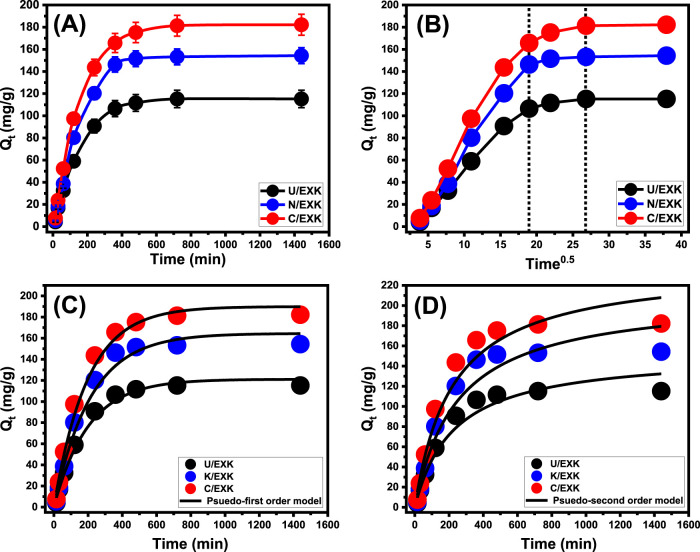
The influence of time frame on the adsorption of SFR by U/EXK, N/EXK, and C/EXK **(A)**, Intra-particle diffusion curves **(B)**, fitting of the SFR uptake with Pseudo-First order model **(C)**, and fitting of the SFR uptake with Pseudo-second order model **(D)**.

An investigation had been performed to follow the adsorption properties of U/EXK, N/EXK, and C/EXK in terms of the duration of SFR elimination. The duration of the test ranged between 15 and 1,440 min. Following the maintenance of the other crucial parameters, like the SFR content (100 mg/L), pH (8), volume (100 mL), temperature (20°C), and dosage (0.4 g/L), the designated influence of time intervals was assessed. The efficacy of U/EXK, N/EXK, and C/EXK throughout the SFR retention processes reveals a substantial rise in both the determined amount of SFR captured and the experimental elimination rates. Furthermore, it is necessary to understand that the duration of the tests plays a major regulating role in the verified increases in the previously mentioned uptake characteristics (Figure 7A). Using U/EXK and C/EXK for 720 min and N/EXK for 480 min results in dramatically enhanced SFR uptake properties. However, there weren’t any discernible changes or enhancements in the speed of SFR removal or the quantities of SFR maintained after the specified interacting durations. Based on earlier research, it can be hypothesized that the U/EXK, N/EXK, and C/EXK adsorbents attained their stable states after the aforementioned periods, which signifies the periods of their equilibrium states during the SFR uptake (Figure 7A). It has been determined that SFR has equilibrium retaining capacities of 115.2 mg/g, 154.3 mg/g, and 182.2 mg/g using U/EXK, N/EXK, and C/EXK, respectively. Powerful enhancements and rises in the extent of SFR elimination and the quantities of SFR retained were observed in the early stages of examination related to the abundance of a substantial quantity of reacting and unbound receptors across the structures of U/EXK, N/EXK, and C/EXK (El-Sherbeeny et al., 2021). The number of vacant receptors drops significantly with longer testing times. This is primarily attributable to the prolonged binding of SFR, which eventually occupies the aforementioned receptors and decreases the total number of unfilled receptors. As a result, after a certain period of time, the speed of SFR binding demonstrated a significant decline. Moreover, the uptake of SFR using U/EXK, N/EXK, and C/EXK showed limited improvement or stable characteristics. By occupying all the binding receptors, the equilibrium stages of U/EXK, N/EXK, and C/EXK can be recognized, and no additional SFR molecules can be adsorbed on their surfaces (Alhalili and Abdelrahman, 2024).

##### 3.2.2.2 Intra-particle diffusion behavior

The analysis of SFR uptake behaviors using U/EXK, N/EXK, and C/EXK might potentially be illustrated by examining their intra-particle diffusion tendencies. The presented curves show three distinctive segments with varying slopes (Figure 7B). The ongoing analysis suggests that the evaluated curves deviate from their original positions, suggesting the existence of many adsorption processes alongside the diffusion pathway of SFR (Abdel Salam et al., 2022; Qada, 2020). The operational processes usually comprise three major phases: 1) the interactions among SFR and the unfilled receptors existing across the exterior interfaces of U/EXK, N/EXK, and C/EXK (boundary); 2) the layered capture of SFR alongside the diffusion characteristics of the SFR; and 3) the impact of saturation level and stabilizing conditions (Lin et al., 2021). The first results derived from these analyses suggest that the main processes that are accountable for binding SFR onto the outside layers of U/EXK, N/EXK, and C/EXK (external adsorption) comprised the most significant pathways identified throughout the whole process (Figure 7B). The efficacy of SFR adsorption at this stage relies on the overall quantities of receptors present at the contact interfaces of U/EXK, N/EXK, and C/EXK (Albukhari et al., 2021). Prolonging the time improved the detection of the new mechanistic step and highlighted the effectiveness of extra-layered adsorption reactions immediately after the entirety of external receptors were completely filled up (Figure 7B) (Lin et al., 2021; Abukhadra et al., 2022b). Furthermore, these additional processes involve the consequences of SFR diffusion behaviors. Upon establishing the equilibrium states, the last SFR uptake mechanistic actions of U/EXK, N/EXK, and C/EXK demonstrate a significant effect. This suggests that all the SFR molecules that were successfully retained have filled up all the accessible binding sites (Abdel Salam et al., 2022; Wang et al., 2024). The removal of SFR during this stage is achieved by molecular interactions and interionic attraction mechanisms (Jiang et al., 2020).

##### 3.2.2.3 Kinetic modeling

The use of adsorption-kinetic models is essential for investigating the effects of time as well as determining whether the adsorption process is controlled by physical processes, mainly mass transfer pathways or chemical pathways (Neolaka et al., 2020). The conventional kinetic principles of pseudo-first order (P.F.) and pseudo-second order (P.S.) mathematical models were used for analyzing the kinetic aspects of SFR-eliminating events using U/EXK, N/EXK, and C/EXK. The PFO model is used to analyze the kinetics of uptake activities across the equilibrium operation in order to clarify the relationship between the rate at which the binding sites get saturated by the analyte and the quantity of empty sites. The PSO model is used to illustrate the relationship between the adsorption capacities of assessed adsorbents as time progresses. By using nonlinear fitting parameters in accordance with the corresponding formulas, the agreement levels of the SFR retention procedures and kinetic principles in relation to the two different hypotheses were assessed. The coefficients of determination (*R* ^2^) and Chi-squared (χ^2^) were used to determine the fitting degrees (Table 1; Figures 7C,D).

**TABLE 1:** The mathematical parameters of the addressed kinetic models.

Models	Parameters	U/EXK	N/EXK	C/EXK
Pseudo-First-order	K_1_ (1/min)	0.995	0.995	0.005
	Qe _(Cal)_ (mg/g)	121.1	164.5	189.9
	R (Zourou et al., 2022)	0.98	0.98	0.97
	X (Zourou et al., 2022)	0.90	1.50	1.18
Pseudo-Second-order	k_2_ (mg/g min)	2.88 × 10^−6^	1.92 × 10^−5^	1.90 × 10^−5^
	Qe _(Cal)_ (mg/g)	153.1	210.1	239.5
	R (Zourou et al., 2022)	0.96	0.96	0.97
	X (Zourou et al., 2022)	1.92	3.09	2.71

The *R* ^2^ values, together with the χ^2^ statistics, indicate that the kinetic properties and principles of the P.F. theory donate a better fit for the adsorption actions of SFR using U/EXK, N/EXK, and C/EXK compared to the assessed P.S. assumption. The measured equilibrium uptake quantities (115.2 mg/g (U/EXK), 154.3 mg/g (N/EXK), and 182.2 mg/g (C/EXK)) closely matched the results obtained from mathematical calculations using the P.F. model (121.1 mg/g (U/EXK), 164.5 mg/g (N/EXK), and 190 mg/g (C/EXK)). The observed consistency provides further confirmation of the previously demonstrated results, which were highlighted during the kinetic assessments regarding the better compatibility of the P.F. model (Table 1). The P.F. theory suggests that the capture of SFR by U/EXK, N/EXK, and C/EXK is mainly controlled by physical mechanisms such as van der Waals forces or electrostatic attraction (Sherlala et al., 2019; Huang et al., 2018). The observed uptake characteristics likewise demonstrate substantial conformance to the P.S. theory, yet the P.F. modeling provides a higher level of agreement. Prior research has shown that common chemical effects such as hydrogen bonds, complexing, and hydrophobic bonds can either enhance or possess a negligible effect on the reduction of SFR by U/EXK, N/EXK, and C/EXK (Abdel Salam et al., 2022; Sherlala et al., 2019). The previously produced chemically bonded SFR layer may serve as the basis for the development of subsequent SFR adsorbing layers by physical processes (Jasper et al., 2020).

#### 3.2.3 Equilibrium studies

##### 3.2.3.1 Effect of concentrations

**FIGURE 8:**
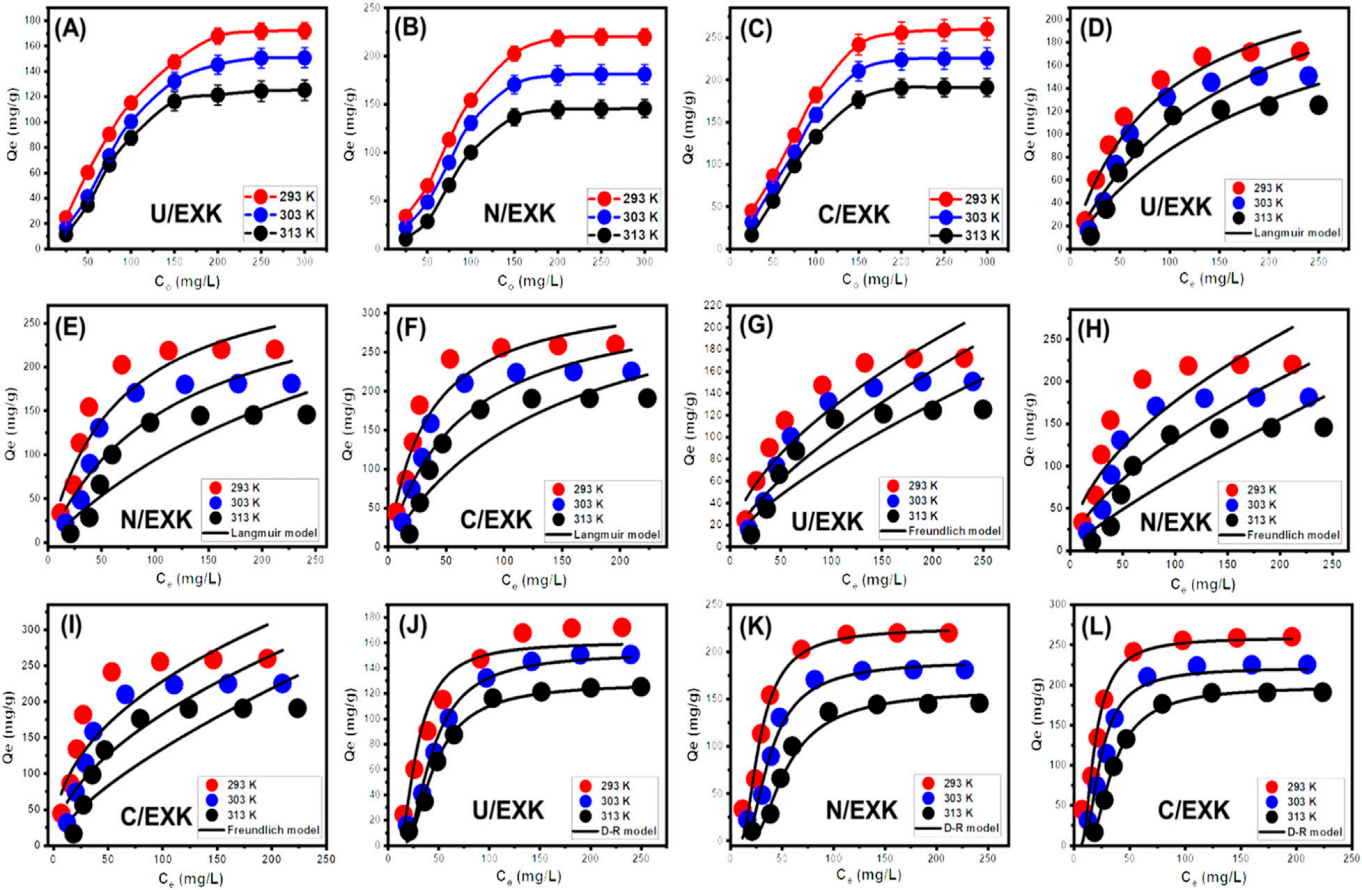
The influence of SFR concetration on its uptake capacity (U/EXK **(A)**, N/EXK **(B)**, and C/EXK **(C)**), fitting of the SFR uptake with Langmuir model (U/EXK **(D)**, N/EXK **(E)**, and C/EXK **(F)**), fitting of the SFR uptake with Freundlich model (U/EXK **(G)**, N/EXK **(H)**, and C/EXK **(I)**), and fitting of the SFR uptake with Langmuir model (U/EXK **(J)**, N/EXK **(K)**, and C/EXK **(L)**).

By analyzing the effects of starting SFR concentrations, the study intended to determine the highest ranges of the SFR removal qualities by U/EXK, N/EXK, and C/EXK as well as corresponding equilibrium situations throughout the assessed range of 25–300 mg/L. The other factors influencing the reduction of SFR were kept constant at defined levels, comprising a total volume of 100 mL, a duration of 24 h, a dosage of 0.4 g/L, and temperatures ranging between 293 K and 313 K. There may be a correlation between the higher SFR levels and the observed increase in the quantities of SFR retained by U/EXK, N/EXK, and C/EXK (Figures 8A–C). The increase in the extent of SFR within a certain volume led to a significant enhancement in the dispersal, driving forces, and migration characteristics of dissolved SFR. This facilitated interactions with a larger number of the functional uptake receptors that are present across U/EXK, N/EXK, and C/EXK surfaces. Hence, the SFR retaining processes conducted by U/EXK, N/EXK, and C/EXK exhibited noteworthy improvements in efficacy with respect to the assessed SFR contents (Ashraf et al., 2022). However, this relationship is only visible within specific constraints on SFR concentrations. Beyond that, increasing the initial concentration of SFR seems to have minimal impact on its uptake by U/EXK, N/EXK, and C/EXK. The identification of the equilibrium stages enables the determination of the accurate maximum retaining effectiveness of SFR. The SFR retaining capacities of U/EXK were 172.2 mg/g at 293 K, 150.8 mg/g at 303 K, and 125.3 mg/g at 313 K (Figure 8A). The equilibrium adsorption qualities with N/EXK were 220.2 mg/g at 293 K, 181.3 mg/g at 303 K, and 145.8 mg/g at 313 K (Figure 8B). The SFR adsorption qualities of C/EXK at various temperatures were 260.2 mg/g at 293 K, 225.6 mg/g at 303 K, and 190 mg/g at 313 K (Figure 8C). The improved uptake properties revealed by C/EXK can be explained by the following reasons: 1) the augmented surface area; 2) the notable improvement in surface reactivity; and 3) a significant rise in the total quantity of binding sites resulting from the enhanced exposure of the active siloxane groups. The reduction in SFR retention encountered by employing U/EXK, N/EXK, and C/EXK at different temperatures indicates that the processes are exothermic.

##### 3.2.3.2 Giles’s classification

The isotherm-based trends for SFR adsorption, employing U/EXK, N/EXK, and C/EXK, have been categorized in accordance with the criteria outlined in Giles’ classification. The investigation revealed that the analyzed curves demonstrated an L-type class. Throughout the elimination operations of SFR utilizing U/EXK, N/EXK, and C/EXK, the isotherm aspects of this class reflect the significant impacts caused by intermolecular attractive reactions (Figures 7A–C). The described activities are intensified by the forceful interaction of SFR, which occurs at the highly reactive surfaces of U/EXK, N/EXK, and C/EXK (Abukhadra et al., 2018). According to the criteria of the L-type class, it was believed that U/EXK, N/EXK, and C/EXK particles would have complete layers of retained SFR developed on their surfaces (Shaban et al., 2017a). Furthermore, the recognized isothermal situations imply that the U/EXK, N/EXK, and C/EXK particulates possess a wide range of vital and effective sites for binding. Moreover, those binding sites exhibit notable affinity towards the SFR molecules, particularly when the starting SFR contents are low.

##### 3.2.3.3 Classic isotherm models

Conventional isotherm studies of adsorption reactions may be used to assess the distribution of dissolved pollutants across the water solution and the particles of adsorbent materials at equilibrium. The clarification of adsorption mechanisms heavily depends on the implementation of traditional equilibrium modeling. The traditional isotherm behaviors offer valuable information about (a) the sorbate’s affinities for the reacting surfaces of adsorbents, (b) the quantities of water-soluble molecules that can potentially be adsorbed by them, and (c) the greatest potential for adsorption. The Langmuir (Figures 8C–E), Freundlich (Figures 8G–I), and Dubinin-Radushkevich (D-R) (Figures 8J–L) equilibrium concepts were used to evaluate the isotherm characteristics of SFR sequestered characteristics utilizing U/EXK, N/EXK, and C/EXK. The non-linear equations corresponding to the aforementioned theories have been provided in Table 2. The level of agreement among the stated equilibrium assumptions of the previously stated models and the practically measured SFR sequestration tendencies was evaluated using non-linear fitting approaches with the respective formulations for these mathematical models. The assessment involved investigating the correlation coefficient (*R* ^2^) in conjunction with the Chi-squared (χ^2^) findings. The examination of *R* ^2^ and χ^2^ indicates that the U/EXK, N/EXK, and C/EXK particulates have SFR adsorbing qualities that correlate more closely with Langmuir’s principles than the Freundlich assumption. This equilibrium behavior (Langmuir isotherm) indicates that the binding affinity of SFR is homogeneous and uniformly distributed throughout the free and activated sites of U/EXK, N/EXK, and C/EXK particulates, forming a single or a monolayer of retained SFR (Sherlala et al., 2019; Huang et al., 2018). However, the Freundlich assumption donates the heterogeneous uptake of the dye molecules in multilayered form. Additionally, investigation proved that U/EXK, N/EXK, and C/EXK particles possess favorable SFR-retaining properties, as evidenced by RL values less than 1 (Albukhari et al., 2021; Wang et al., 2024). The highest adsorption capacities (Q_max_) of SFR by U/EXK, were 287.3 mg/g at 293 K, 262.23 mg/g at 303 K, and 257.2 mg/g at 313 K (Table 2). The calculated values for N/EXK were 331.3 mg/g at 293 K, 323 mg/g at 303 K, and 312.8 mg/g at 313 K (Table 2). The outcomes obtained for C/EXK were 364.4 mg/g at 293 K, 332.7 mg/g at 303 K, and 329 mg/g at 313 K.

**TABLE 2:** The mathematical parameters of the addressed classic isotherm models.

Models	Parameters	U/EXK			N/EXK			C/EXK		
		293 K	303 K	313 K	293 K	303 K	313 K	293 K	303 K	313 K
Langmuir	Q_max_ (mg/g)	287.3	262.2	257.2	331.3	323.0	312.8	364.4	332.7	329.1
	b (L/mg)	0.006	0.011	0.005	0.003	0.015	0.008	0.007	0.029	0.015
	R (Zourou et al., 2022)	0.92	0.95	0.90	0.87	0.92	0.89	0.88	0.95	0.91
	X (Zourou et al., 2022)	3.18	2.52	4.01	4.13	2.89	3.55	4.38	2.63	3.12
Freundlich	1/n	0.572	0.693	0.733	0.535	0.635	0.836	0.437	0.533	0.698
	k_F_ (mg/g)	9.03	4.07	2.68	15.02	7.01	1.85	30.50	15.70	5.41
	R (Zourou et al., 2022)	0.88	0.87	0.84	0.83	0.82	0.82	0.84	0.82	0.90
	X (Zourou et al., 2022)	4.30	4.67	5.17	5.27	5.92	6.18	5.33	5.67	3.42
Dubinin-Radushkevich	β (mol (Zourou et al., 2022)/KJ (Zourou et al., 2022))	0.0055	0.0058	0.0061	0.0072	0.0073	0.0074	0.0060	0.0064	0.0066
	Q_m_ (mg/g)	160.6	152.1	128.1	225.6	190.6	160.2	259.3	221.9	198.5
	R (Zourou et al., 2022)	0.96	0.97	0.98	0.92	0.94	0.96	0.90	0.96	0.99
	X (Zourou et al., 2022)	2.37	1.99	1.03	2.04	1.53	1.38	3.94	2.25	0.11
	E (KJ/mol)	9.51	9.22	8.90	8.30	8.26	8.19	9.12	8.80	8.65

The equilibrium features of the D-R theory provide a thorough comprehension of the energy fluctuations displayed by U/EXK, N/EXK, and C/EXK particulates during SFR scavenging operations, regardless of the particle’s level of homogeneity or heterogeneity (Dawodu and Akpomie, 2012). Analyzing the D-R simulation outcomes has significance for calculating the Gaussian energy (E) and understanding the fundamental mechanisms (chemical or physical). The adsorption reactions exhibit energy levels below 8 kJ/mol, within 8–16 kJ/mol, and above 16 kJ/mol, which mainly correspond to strong physical, predominantly diminished chemical-based, or a mix of chemical alongside physical and predominantly strong chemical-based mechanisms, respectively (Wang et al., 2024). The measured values of E corresponding to SFR-retaining reactions by U/EXK, N/EXK, and C/EXK are inside the specified energy range (8–16 kJ/mol) for co-operated chemical and physical activities.

##### 3.2.3.4 Advanced isotherm modeling

**FIGURE 9:**
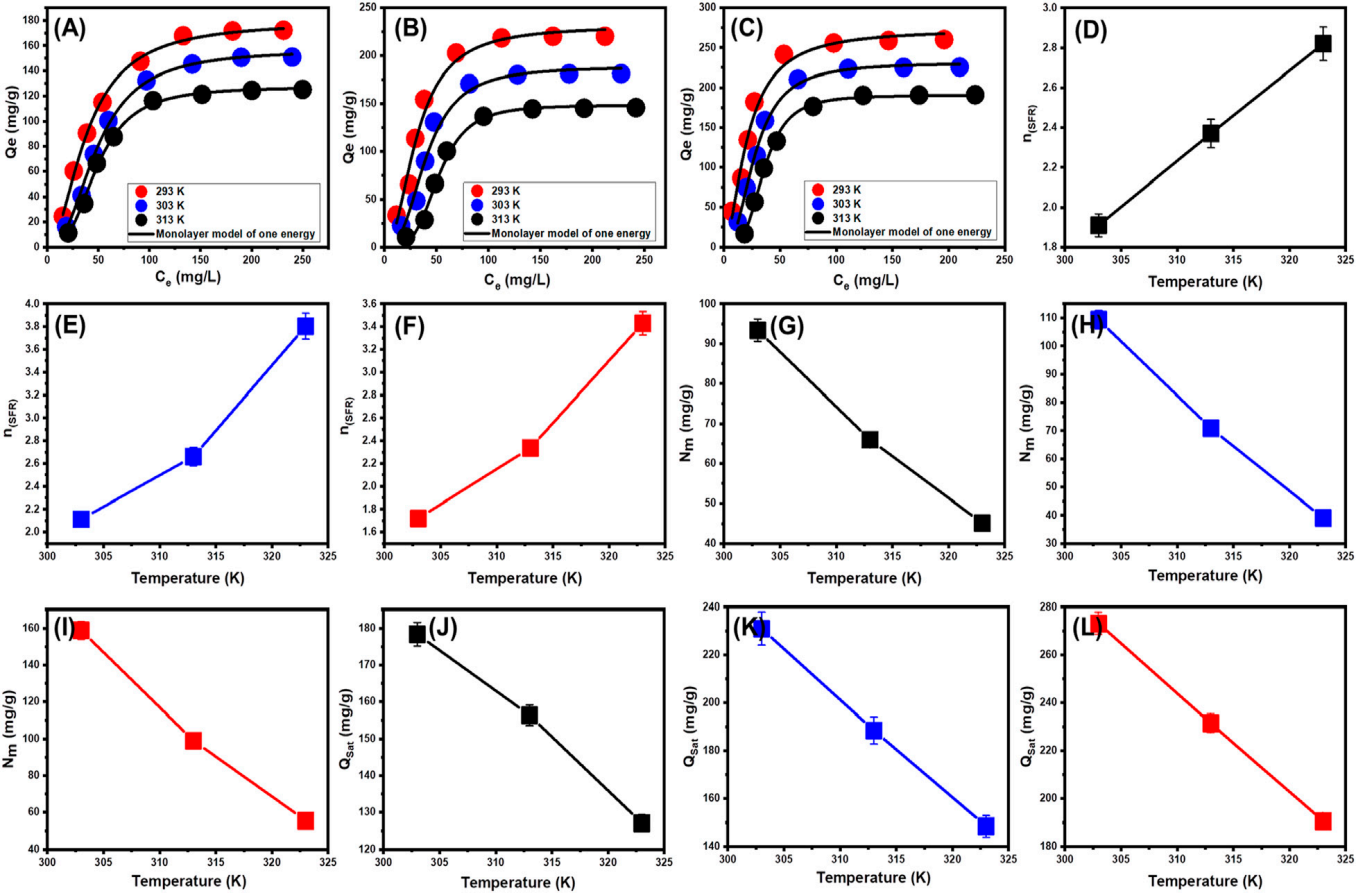
Fitting of the SFR uptake with Monolayer model of single energy site (U/EXK **(A)**, N/EXK **(B)**, and C/EXK **(C)**), changes in the number of adsorbed SFR molecules per each site (U/EXK **(D)**, N/EXK **(E)**, and C/EXK **(F)**), changes in the uptake sites density (U/EXK **(G)**, N/EXK **(H)**, and C/EXK **(I)**), and changes in the saturation uptake capacities (U/EXK **(J)**, N/EXK **(K)**, and C/EXK **(L)**).

The classic isotherm models as Langmuir model give no remarkable physical significance about the adsorption processes and cannot describe the relation between the different physicochemical parameters during the adsorption process (Dhaouadi et al., 2021). Therefore, the advanced isotherm models based on the basic s of statistical physics theories were recommended in the later periods. Utilizing statistical physics approaches to simulate the equilibrium of adsorption activities may provide an in-depth analysis of the specific qualities of adsorption processes. These mathematical simulations evaluate the interactions between soluble contaminants and exterior active functionalities as reacting receptors across the interfaces of solid materials as adsorbing agents. The mathematical models used in this study comprise a number of computation variables that successfully depict the fundamental mechanisms that involve both energetic and steric factors. The models develop steric aspects such as the receptor site occupation (n) by SFR individually, the overall number of sites occupied by SFR throughout the U/EXK, N/EXK, and C/EXK interfaces (N_m_), and the maximum uptake effectiveness of SFR by U/EXK, N/EXK, and C/EXK when they are totally saturated (Q_sat_). The energetic characteristics consist of internal energy (E_int_), entropy (Sa), retaining energy (E), and free enthalpy (G). The evaluation of these theories included the application of non-linear regression analyses using the represented equations derived for the relevant models. The earlier investigation was effectively performed by employing multivariable nonlinear regression analyses in combination with the Levenberg-Marquardt iterative method. The obtained levels of fitness were subsequently utilized to assess and characterize the adsorption tendencies of SFR via U/EXK, N/EXK, and C/EXK employing the highly fitted model (monolayer model of a single energetic site (Figures 9A–C)). Table 3 provides the computed parameters along with the fitting factors.

**TABLE 3:** The mathematical parameters of the studied advanced isotherm model.

		293 K	303 K	313 K
U.EXK	R (Zourou et al., 2022)	0.99	0.99	0.99
	X (Zourou et al., 2022)	0.09	0.19	0.14
	n	1.91	2.37	2.82
	Nm (mg/g)	93.40	65.98	45.07
	Q_Sat_ (mg/g)	178.4	156.4	127.1
	C1/2 (mg/L)	38.4	48.6	48.8
	ΔE (kJ/mol)	−8.88	−9.78	−10.10
N.EXK	R (Zourou et al., 2022)	0.98	0.98	0.98
	X (Zourou et al., 2022)	1.75	1.61	1.20
	n	2.11	2.66	3.80
	N_m_ (mg/g)	109.3	70.8	39.0
	Q_Sat_ (mg/g)	231.0	188.4	148.4
	C1/2 (mg/L)	31.2	40.4	52.3
	ΔE (kJ/mol)	−8.38	−9.31	−10.29
C.EXK	R (Zourou et al., 2022)	0.98	0.99	0.99
	X (Zourou et al., 2022)	1.34	0.23	0.10
	n	1.72	2.33	3.43
	N_m_ (mg/g)	158.8	99.0	55.5
	Q_Sat_ (mg/g)	273.2	231.4	190.5
	C1/2 (mg/L)	20.9	27.7	35.4
	ΔE (kJ/mol)	−7.40	−8.36	−9.29

###### 3.2.3.4.1 Steric properties

####### 3.2.3.4.1.1 Number of adsorbed SFR (n) per each site

The numerical findings of the n _(SFR)_ factor provide adequate proof of the arrangement behavior of the adsorbed SFR ions occupying the exterior surfaces of U/EXK, N/EXK, and C/EXK. This includes both the vertical as well as the horizontal orientations. Additionally, these outcomes are significant in understanding the mechanisms that regulate the reactions, such as multi-docking or multi-interactions. The capture of one SFR ion via multiple uptake sites is one of the reactions that are most significantly impacted by multi-anchorage or multi-docking operations. The retention reactions with values less than one are associated with the ions’ horizontal orientation. Conversely, activities that display values above 1 indicate the retaining of SFR within non-parallel geometries together with a vertical orientation. The uptake processes in such systems are mainly mediated by multi-molecular pathways, whereby a single site captures several dye ions (Wang et al., 2024; Mobarak et al., 2021). The determined values of n, which represent the number of bound SFR by a single site of U/EXK, range from 1.91 to 2.37 (Figure 9D). The quantity of SFR molecules maintained by a single site throughout the interfaces of N/EXK (with a range of n _(SFR)_ = 2.11–3.8) (Figure 9E) and C/EXK (with a range of n _(SFR)_ = 1.7–3.4) (Figure 9F) was found to be consistently greater than 1 as determined for U/EXK. As a result, the SFR dye was adsorbed by multi-molecular mechanisms. Each retaining site within the U/EXK, N/EXK, and C/EXK particulates had the ability to accommodate multiple ions that were organized in vertical configurations with distinct non-parallel characteristics. The individual receptors within the exterior surface of U/EXK can hold up to 3 SFR ions; however, each binding site through the external surfaces of N/EXK and C/EXK is capable of holding 4 ions. This suggests considerable changes in the exterior properties of scrubbed kaolinite depending on the used methods and the specific interspersed reagent. The n _(SFR)_ computations for U/EXK, N/EXK, and C/EXK show a significant rise via temperature from 293 K to 313 K. The behavior that has been noticed may be attributed to the presumed increase in the SFR’s aggregation qualities during its adsorption by U/EXK, N/EXK, and C/EXK at elevated temperatures (Ashraf et al., 2022). This also indicates the presence of thermal activation mechanisms prior to the retention of SFR utilizing U/EXK, N/EXK, and C/EXK (Yang et al., 2022; Dhaouadi et al., 2021).

####### 3.2.3.4.1.2 Occupied active sites density (N_m_)

The density of SFR-occupied sites (Nm _(SFR)_) of U/EXK, N/EXK, and C/EXK may accurately indicate the total amount of free and functional binding receptors onto the exteriors of their particles across the overall reaction (Figures 8E–H; Table 4). U/EXK’s estimate indicates that the amounts of Nm _(SFR)_ at various temperatures are 93.4 mg/g (293 K), 65.9 mg/g (303 K), and 45.1 mg/g (313 K) (Figure 9G; Table 3). The quantities showed a notable rise after the exfoliating process employing KNO_3_ (N/EXK), reaching 109.3 mg/g at 293 K, 70.8 mg/g at 303 K, and 39.4 mg/g at 313 K (Figure 9H; Table 3). The degree of improvement increased greatly following the exfoliation process employing the CTAB embedding agent, resulting in quantities of 158.8 mg/g (293 K), 99 mg/g (303 K), and 55.5 mg/g (313 K) (Figure 9I; Table 3). The results presented provide reliable proof of a significant increase in the number of existing receptors after exfoliating with CTAB (C/EXK) in comparison with U/EXK and N/EXK. The increase in surface area, coupled with the improved surficial reactivity and the greater exposure for the influential siloxane groups, leads to an improved contact and interaction interface among SFR ions and the C/EXK surface. Regarding their temperature responses, the Nm _(SFR)_ values for U/EXK, N/EXK, and C/EXK reveal temperature-dependent reversible changes. This aligns with the tracked responses of n _(SFR)_, as the higher aggregation affinities of SFR dye result in a decrease in the overall number of occupied receptors as well. Furthermore, the temperature significantly affects the activity degrees of the existing receptors that were involved in the reaction (Yang et al., 2022; Sellaoui et al., 2020).

**TABLE 4:** Comparison between the adsorption properties of EXK structures and other studied structures in literature.

Adsorbent	q_max_ (mg/g)	Reference
Ppy NF/Zn-Fe LDH	63.4	Mohamed et al. (2018)
Glass-MCM-48	62.5	Abukhadra and Shaban (2019)
Activated carbon	576	Kumar and Sivanesan (2005)
MCM-41	68.8	Kaur et al. (2015)
MgO-FLG-FE	201.1	Reddy et al. (2018)
CuO-NP	189.5	Vidovix et al. (2021)
N/porous graphite	20.66	Shaban et al. (2017b)
Sepiolite	233.81	Barhdadi et al. (2024)
Ferruginous kaolinite	59.3	Debnath et al. (2017)
Magnetic clay	18.48	Abukhadra et al. (2020b)
Raw kaolinite	14.37	This study
U/EXK	178.4	This study
N/EXK	231	This study
C/EXK	273.2	This study

####### 3.2.3.4.1.3 Adsorption capacity at the saturation state of (Q_sat_)

The saturation adsorption properties of U/EXK, N/EXK, and C/EXK (Q_sat_) demonstrate the predicted maximum quantities of sequestered SFR with the greatest level of tolerance. The values of Q_sat_ are controlled by two main variables: the specified density of the loaded sites (Nm) and the total number of SFR ions captured by a single receptor (n). U/EXK, a potential adsorbent for SFR, demonstrates Q_sat_ values of 178.4 mg/g at 293 K, 156.4 mg/g at 303 K, and 127.1 mg/g at 313 K (Figure 9J; Table 3). The utilization of N/EXK exhibits hypothesized maximum capacities of 231 mg/g (at 293 K), 188.4 mg/g (at 303 K), and 148.4 mg/g (at 313 K) (Figure 9K; Table 3). The application of C/EXK demonstrated improved effectiveness, exhibiting values of 273.2 mg/g at 293 K, 231.4 mg/g at 303 K, and 190.5 mg/g at 313 K (Figure 9L; Table 3). The exothermic properties of SFR retaining operations using U/EXK, N/EXK, and C/EXK can potentially be noticed by the adverse effect of temperature. This implies that the effect of thermal collisions rises with retaining temperature and reduces the effectiveness of SFR binding (Mobarak et al., 2021). Furthermore, the observed characteristics of Q_sat_ associated with temperature fluctuations suggest a resemblance to the specified tendencies of Nm instead of n. This proves that the effectiveness of SFR adsorption depends on the quantities of the binding receptors instead of the capacity of every single binding site.

###### 3.2.3.4.2 Energetic properties

####### 3.2.3.4.2.1 Adsorption energy

The energy variations (ΔE) encountered throughout the uptake activities of SFR may accurately reveal the underlying mechanisms, whether they are related to physical or chemical behaviors. The physical processes exhibit energy beneath 40 kJ/mol, while the chemical paths have energy exceeding 80 kJ/mol. Adsorption energy can be utilized to categorize a variety of physically occurring mechanistic processes. The physical interactions reported include coordinating exchange (40 kJ/mol), bonding of hydrogen (<30 kJ/mol), dipole bonding (2–29 kJ/mol), electrostatic attractions (2–50 kJ/mol), van der Waals forces (4–10 kJ/mol), and hydrophobic bonding (5 kJ/mol) (Ashraf et al., 2022; Ali et al., 2021). The theoretical determination of the SFR elimination energies (ΔE) was accomplished using Equation 5, considering the soluble state of SFR inside water (S), gas constant (R = 0.008314 kJ/mol.K), SFR contents throughout half saturation situations of U/EXK, N/EXK, and C/EXK, and actual temperature (T) (Dhaouadi et al., 2020).

$$∆E=RT\;\ln\left(\frac{S}{C}\right)$$ (5)

The energy measurements for SFR retention, as determined for U/EXK and N/EXK, vary from −8.8 and −8.38 kJ/mol to −10.11 and −10.29 kJ/mol, respectively (Table 3). The estimated values for C/EXK varied between −7.4 and −9.29 kJ/mol (Table 4). Hence, the key mechanisms responsible for the uptake of SFR via U/EXK, N/EXK, and C/EXK were physical processes, including dipole bonding (2–29 kJ/mol), van der Waals forces (4–10 kJ/mol), electrostatic attractions (2–50 kJ/mol), and hydrogen bonding (<30 kJ/mol). Additionally, the observed negative indications of the expected E values during the capture of SFR are consistent with the prior experimental results describing the exothermic characteristics of these reactions.

###### 3.2.3.4.3 Thermodynamic functions

####### 3.2.3.4.3.1 Entropy

The entropy (Sa) corresponding to the SFR retention processes by U/EXK, N/EXK, and C/EXK provides an obvious illustration of the order and disorder characteristics of the outermost surfaces of their nanostructures when subjected to different levels of dye ions, in addition to the specified reaction temperature. The Sa characteristics were illustrated by implementing the results obtained from Equation 6, including the previously established measurements of Nm and n, in addition to the predicted contents of SFR throughout the half-saturation states of U/EXK, N/EXK, and C/EXK (C1/2).

$$\frac{S_{a}}{K_{B}}=Nm\left\{\ln\left(1+{\left(\frac{C}{C_{\frac{1}{2}}}\right)}^{n}\right)-n{\left(\frac{C}{C_{\frac{1}{2}}}\right)}^{n}\;\frac{\ln\left(\frac{C}{C_{\frac{1}{2}}}\right)}{1+{\left(\frac{C}{C_{\frac{1}{2}}}\right)}^{n\;}}\;\right\}$$ (6)

**FIGURE 10:**
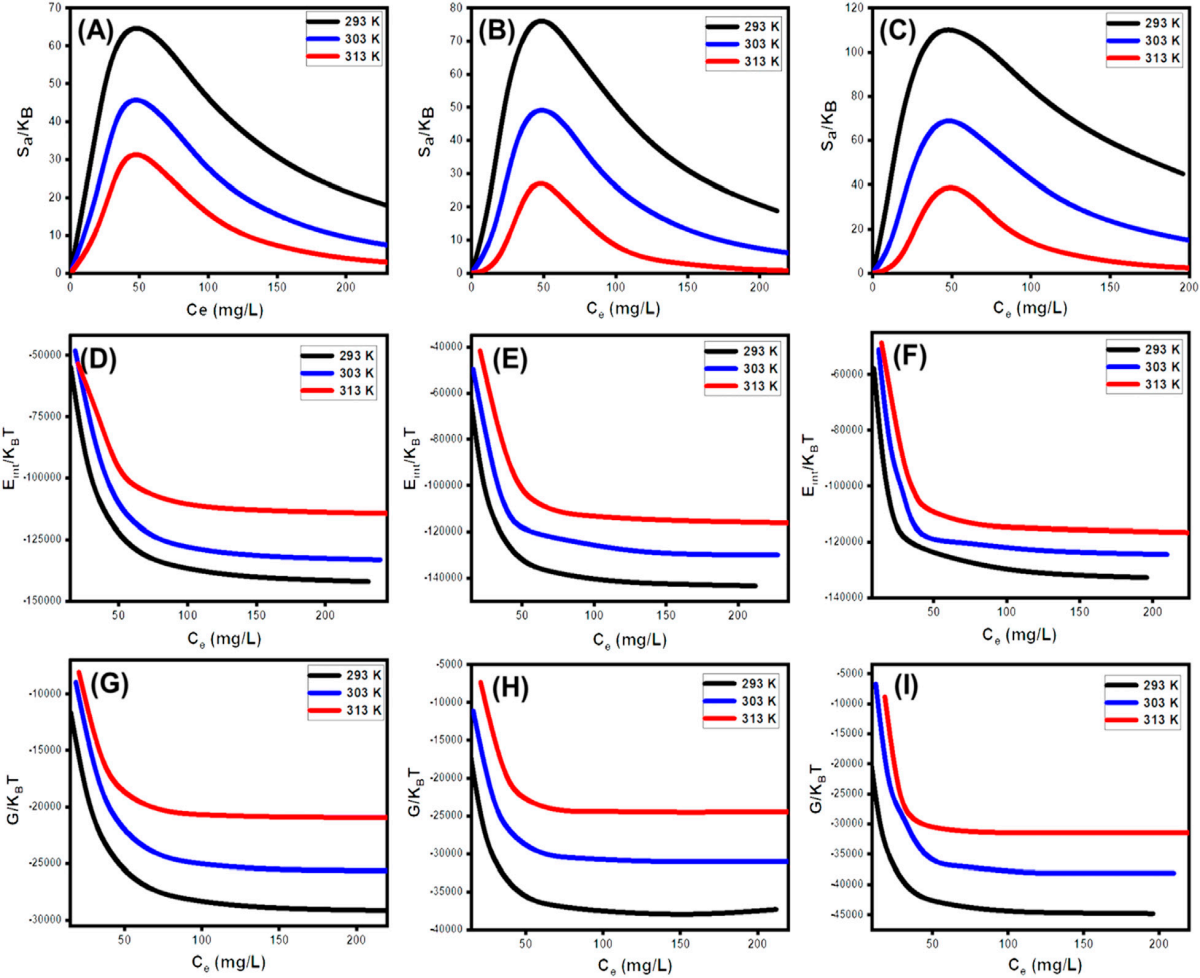
Changes in the thermodynamic functions during the upatke of SFR including entropy (U/EXK **(A)**, N/EXK **(B)**, and C/EXK **(C)**), internal energy (U/EXK **(D)**, N/EXK **(E)**, and C/EXK **(F)**), and enthalpy (U/EXK **(G)**, N/EXK **(H)**, and C/EXK **(I)**).

From the observed curves, it is evident that the entropy levels (Sa) experience a significant drop when SFR gets captured by U/EXK, N/EXK, and C/EXK, especially at elevated SFR concentrations (Figures 10A–C). This trend indicates a notable decrease in the disorder characteristics that characterize the interfaces of U/EXK, N/EXK, and C/EXK with increasing examined SFR extents. The entropy characteristics also facilitate the successful docking of SFR onto the unoccupied and influential interacting sites of U/EXK, N/EXK, and C/EXK, regardless of the presence of small concentrations of SFR (Sellaoui et al., 2020; Dhaouadi et al., 2020). The maximum magnitudes of entropy were noted when SFR was captured by U/EXK at equilibrium levels of 38.8 mg/L (293 K), 45.6 mg/L (303 K), and 48.4 mg/L (313 K) (Figure 10A). The equilibrium values for the greatest entropy during the elimination of SFR using N/EXK are 38.3 mg/L at a temperature of 293 K, 47.7 mg/L at a temperature of 303 K, and 48.4 mg/L at a temperature of 313 K (Figure 10B). The employing of C/EXK demonstrates the highest degree of entropy at SFR concentrations of 27.2 mg/L (at 293 K), 36.5 mg/L (at 303 K), and 46.8 mg/L (at 313 K) (Figure 10C). The equilibrium levels closely approximate the concentrations estimated for the half-saturation stages of U/EXK, N/EXK, and C/EXK. As a result, additional SFR ions are unable to dock with the remaining accessible binding receptors. Additionally, the considerable decreases in the assessed entropy degrees suggest an extensive decrease in the quantity of accessible sites, as well as a noticeable decrease in the mobility and diffuse characteristics of the SFR ions (Sellaoui et al., 2016).

####### 3.2.3.4.3.2 Internal energy and free enthalpy

The study assessed the internal energy (E_int_) correlated with the binding reactions of U/EXK, N/EXK, and C/EXK, alongside the qualities of free enthalpy (G) and the manner in which they are affected by changes in dye contents and operating temperature. The evaluation was conducted using values obtained from Equations 7, 8, which were computed using the earlier established Nm, n, and C1/2, along with the translation partition (Zv) (Dhaouadi et al., 2021).

$$\frac{E_{\mathrm{int}}}{K_{B}T\;}=n\;N_{m}\left[\left(\;\frac{{\left(\frac{C}{C_{1/2}}\right)}^{n}\;\ln\left(\frac{C}{Z_{v}}\right)}{1+{\left(\frac{C}{C_{1/2}}\right)}^{n}}\right)-\left(\;\frac{n\ln\left(\frac{C}{C_{1/2}}\right)\;{\left(\frac{C}{C_{1/2}}\right)}^{n}}{1+{\left(\frac{C}{C_{1/2}}\right)}^{n}}\right)\right]$$ (7)

$$\frac{G}{K_{B}T\;}=n\;N_{m}\left(\frac{\ln\left(\frac{C}{Z_{v}}\right)}{1+{\left(\frac{C_{1/2}}{C}\right)}^{n}}\right)$$ (8)

The calculated values of E_int_ with respect to SFR retention processes via U/EXK, N/EXK, and C/EXK possess negative signs, and these findings reveal a significant decrease whenever the temperature is increased from 293 K to 313 K (Figures 1D–F). This validates the spontaneous alongside exothermic characteristics of the SFR elimination processes via U/EXK, N/EXK, and C/EXK. Similar behaviors and properties have been identified for the described levels and behaviors of enthalpy (Figures 10G–I). The G results have negative signs and demonstrate a reversible correlation with the practical retention temperature. This suggests a decrease in the feasibility features and confirms the spontaneity and exothermic behaviors of the SFR sequestration using U/EXK, N/EXK, and C/EXK (Figures 10K,L).

#### 3.2.4 Recyclability

**FIGURE 11:**
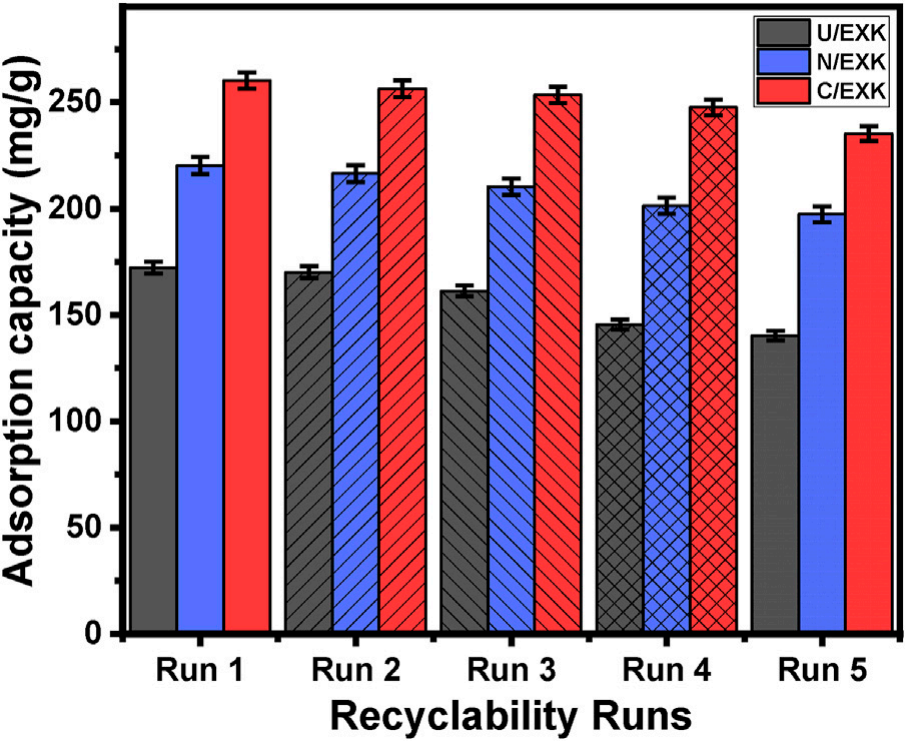
The recyclability properties of the synthetic exfoliated products during the adsorption of SFR.

The efficacy of U/EXK, N/EXK, and C/EXK as adsorption agents for SFR has been investigated, with a focus on their recycling potential, a crucial factor in assessing their feasibility for industrial and realistic uses. The U/EXK, N/EXK, and C/EXK structures underwent thorough rinsing with distilled water throughout a period of 10 min, and this process had been repeated multiple times. The U/EXK, N/EXK, and C/EXK had been subsequently dried at a temperature of 60°C for a period of 10 h to enhance their suitability for use in further SFR purification cycles. The recyclability experiments were carried out under controlled conditions, including a pH of 8, a dosage of 0.40 g/L, a duration of 24 h, a volume of 100 mL, SFR levels of 100 mg/L, and a temperature of 293 K. The outcomes of the five experimental iterations performed to evaluate the utilization of U/EXK, N/EXK, and C/EXK as adsorbents exhibit steady and impressive effectiveness in capturing SFR, together with notable stability and satisfactory reusability characteristics (Figure 11). The U/EXK material exhibited notable recyclability, achieving removal effectiveness of 172.2 mg/g (Run 1), 170.1 mg/g (Run 2), 161.3 mg/g (Run 3), 145.4 mg/g (Run 4), and 140.3 mg/g (Run 5). The purification process of SFR resulted in elimination capacities of 220.2 mg/g (Run 1), 216.4 mg/g (Run 2), 210.3 mg/g (Run 3), 201.4 mg/g (Run 4), and 197.4 mg/g (Run 5) while recycling N/EXK. The results obtained by employing C/EXK during the recycling experiments are as follows: 260.2 mg/g (Run 1), 256.4 mg/g (Run 2), 253.4 mg/g (Run 3), 247.6 mg/g (Run 4), and 235.2 mg/g (Run 5).

#### 3.2.5 Comparison study

The recognized adsorption capacities of the three forms of exfoliated kaolinite (U/EXK, N/EXK, and C/EXK) were compared with the reported adsorption capacities of other investigated adsorbents in the literature (Table. 4). The exfoliated products displayed higher adsorption properties for safranin dye as compared to raw kaolinite and other different species of clay-based adsorbents, including ferruginous kaolinite, magnetic clay, and sepiolite, in addition to other species of adsorbents such as LDH, mesoporous silica, graphite, CuO, MgO, and activated carbon.

## 4 Conclusion

Kaolinite mineral was exfoliated successfully into monolayer using three different intercalating agents (urea (U/EXK), KNO_3_ (N/EXK), and CTAB (C/EXK)). The applied characterization techniques confirmed effective impact for the applied agent and exfoliation technique on the properties of the obtained monolayers. The structural, morphological, and textural results reflected the highest exfoliation degree of C/EXK as compared to U/EXK and N/EXK. C/EXK also exhibits the best uptake performance of SFR (273.2 mg/g) as compared to 231 mg/g for N/EXK and 178.4 mg/g for U/EXK. This is correlated with its highest surface area (55.7 m^2^/g) and effective sites density (Nm _(SFR)_ = 158.8 mg/g) in contrast to U/EXK and N/EXK. The three products display no significant variation in the controlling mechanism based on the adsorption energy (ΔE < 40 kJ/mol). The regulating mechanisms are mainly physical, spontaneous, and exothermic reactions involving dipole bonding, van der Waals forces, and hydrogen bonding. Based on the optimization conditions, the determined adsorption capacities, and recyclability values, the synthetic structures can be effectively applied as reliable adsorbents during the realistic removal of safranin-O dye from industrial wastewater that is related to the discharges of staining industries as well as the healthcare sector and the packaging of foods.

## Data Availability

The original contributions presented in the study are included in the article/Supplementary Material, further inquiries can be directed to the corresponding authors.
