# Supplementary material for: Physicochemical, steric, and energetic characterization of kaolinite based silicate nano-sheets as potential adsorbents for safranin basic dye: effect of exfoliation reagent and techniques
Source: Front Chem. 2024 Oct 18;12:1455838. doi: 10.3389/fchem.2024.1455838 (PMC11528917; doi:10.3389/fchem.2024.1455838)
Supplement: Supplementary file 1 [file Table1.DOCX]

***Supporting Information***

**Physicochemical, steric, and energetic characterization of kaolinite based silicate nano-sheets as potential adsorbents for safranin basic dye: Effect of exfoliation reagent and techniques**

**Samar Mohamed Ali^1,2^; Reham A. Mohamed^1^; *Ahmed A. Abdel-Khalek^1^*, Ashour M. Ahmed^3^٭; Noof A. Alenazi^4^, M. Abdel Rafea^3^, Mostafa R. Abukhadra٭^2,5^, Stefano Bellucci٭^6^**

^1^Department of Chemistry, Faculty of Science, Beni-Suef University, 62514 Beni-Suef City, Egypt

^2^Materials Technologies and their Applications Lab, Geology Department, Faculty of Science, Beni-Suef University, Beni-Suef City, Egypt

^3^Physics Department, College of Science, Imam Mohammad Ibn Saud Islamic University (IMSIU), Riyadh 11623, Kingdom of Saudi Arabia

^4^Department of Chemistry, College of Science and Humanities in Al-Kharj, Prince Sattam bin Abdulaziz University, Al-kharj 11942, Saudi Arabia

^5^Geology Department, Faculty of Science, Beni-Suef University, Beni-Suef City, Egypt.

^6^INFN-Laboratori Nazionali di Frascati, Vie. E. Fermi 54, 00044 Frascati, Italy

Corresponding author٭: [Abukhadra89@Science.bsu.edu.eg](mailto:Abukhadra89@Science.bsu.edu.eg) (M.R. A.); asmmohamed@imamu.edu.sa (A.M.A); [bellucci@lnf.infn.it](mailto:bellucci@lnf.infn.it) (S.B.)

)

**Content**

1. Nonlinear equations of kinetic, classic isotherm, and advanced isotherm models.…………………...S2

**(S1)**

**Table S1.** Nonlinear equations of kinetic, classic isotherm, and advanced isotherm models

| Kinetic models | | |
| --- | --- | --- |
| Model | **Equation** | **Parameters** |
| Pseudo-first-order | $Q_{t}=Q_{e} (1-e^{{-k}_{1}.t})$ | Q_t_ (mg/g) is the adsorbed ions at time (t), and K_1_ is the rate constant of the first-order adsorption (1/min) |
| Pseudo-second-order | $Q_{t}=\frac{Q_{e}^{2}k_{2}t}{1+Q_{e}k_{2}t}$ | Qe is the quantity of adsorbed ions after equilibration (mg/g), and K_2_ is the model rate constant (g/mg min). |
| Classic Isotherm models | | |
| Model | **Equation** | **Parameters** |
| Langmuir | $Q_{e}=\frac{Q_{max} bC_{e}}{(1+bC_{e})}$ | *C_e_* is the rest ions concentrations (mg/L), *Q_max_* is the theoritical maximum adsorption capacity (mg/g), and *b* is the Langmuir constant (L/mg) |
| Freundlich | $Q_{e}=K_{f}C_{e}^{1/n}$ | K_F_ (mg/g) is the constant of Freundlich model related to the adsorption capacity and n is the constant of Freundlich model related to the adsorption intensities |
| Dubinin–Radushkevich | $Q_{e}=Q_{m}e^{-\betaɛ^{2}}$ | β (mol^2^/KJ^2^) is the D-R constant, ɛ (KJ^2^/mol^2^) is the polanyil potential, and Q_m_ is the adsorption capacity (mg/g) |
| Advanced isotherm models | | |
| Model | **Equation** | **Parameters** |
| Monolayer model with one energy site (Model 1) | $Q=nN_{o} =\frac{nN_{M}}{1+{(\frac{C1/2}{C})}^{n}}=\frac{Q_{o}}{1+{(\frac{C1/2}{C})}^{n}}$ | Q is the adsorbed quantities in mg/g  n is the number of adsorbed ion per site  Nm is the density of the effective receptor sites (mg/g)  Q_o_ is the adsorption capacity at the saturation state in mg/g  C1/2 is the concentration of the ions at half saturation stage in mg/L  C1 and C2 are the concentrations of the ions at the half saturation stage for the first active sites and the second active sites, respectively  n1 and n2 are the adsorbed ions per site for the first active sites and the second active sites, respectively |
| Monolayer model with two energy sites (Model 2) | $Q=\frac{n_{1}N_{1M}}{1+{(\frac{C_{1}}{C})}^{n_{1}}}+\frac{n_{2}N_{2M}}{1+{(\frac{C_{2}}{C})}^{n_{2}}}$ |  |
| Double layer model with one energy site (Model 3) | $Q=Q_{o}\frac{({\frac{C}{C1/2})}^{n}+2({\frac{C}{C1/2})}^{2n}}{1+({\frac{C}{C1/2})}^{n}+({\frac{C}{C1/2})}^{2n}}$ |  |
| Double layer model with two energy sites (Model 3) | $Q=Q_{o}\frac{({\frac{C}{C1})}^{n}+2({\frac{C}{C2})}^{2n}}{1+({\frac{C}{C1})}^{n}+({\frac{C}{C2})}^{2n}}$ |  |

**(S2)**
